# Consumer Participation and Sense of Choice in Pricing: A Psychosocial Pathway to Enhanced Mental Well-Being and Quality of Life

**DOI:** 10.1192/j.eurpsy.2026.11716

**Published:** 2026-07-31

**Authors:** M. Ekhtera, M. Oveysikian

**Affiliations:** 1 Management, University of Tehran; 2 Psychiatry, University of Rehabilitation and Social Health Sciences, Tehran, Iran, Islamic Republic Of

## Abstract

**Introduction:**

Traditional markets position consumers as passive recipients of fixed prices, limiting their sense of control and engagement—key elements for psychological well-being. Behavioral psychiatry suggests that everyday experiences involving agency and self-determination can activate mechanisms similar to therapeutic empowerment.

Participatory pricing models, where individuals set or negotiate prices, introduce autonomy into routine economic contexts. Autonomy and perceived control are fundamental psychological needs linked to well-being and quality of life. This study examines whether allowing consumers to determine product prices enhances mental well-being through increased autonomy

**Objectives:**

The study tested whether self-determined pricing enhances autonomy and well-being, and whether autonomy mediates this link—suggesting that everyday economic choices can activate psychological empowerment.

**Methods:**

An online controlled experiment was conducted with 60 adults (18–45 years). Participants were randomly assigned to either a fixed-price or self-determined pricing condition. Both viewed identical product descriptions differing only in pricing procedure.

They completed two validated scales: a 4-item Perceived Autonomy Scale (adapted from the Basic Psychological Needs Scale) and the 7-item Warwick–Edinburgh Mental Well-Being Scale.. Independent-samples t-tests and a simple mediation analysis (PROCESS Model 4) tested whether pricing condition (0 = fixed, 1 = self-pricing) affected well-being through autonomy.

**Results:**

Participants in the self-determined pricing condition reported significantly higher perceived autonomy (M = 5.8, SD = 0.7) than those in the fixed-price condition (M = 4.9, SD = 0.9), t(58) = 3.92, p < .001. Similarly, mental well-being scores were higher in the self-pricing group (t(58) = 2.71, p = .009). Mediation analysis revealed a significant indirect effect of pricing condition on well-being through perceived autonomy (β = 0.23, 95% CI [0.09, 0.39]), indicating partial mediation. The findings suggest that allowing individuals to participate in decision-making activates autonomy-related processes, leading to transient improvements in positive mood and perceived quality of life.

**Image 1:**
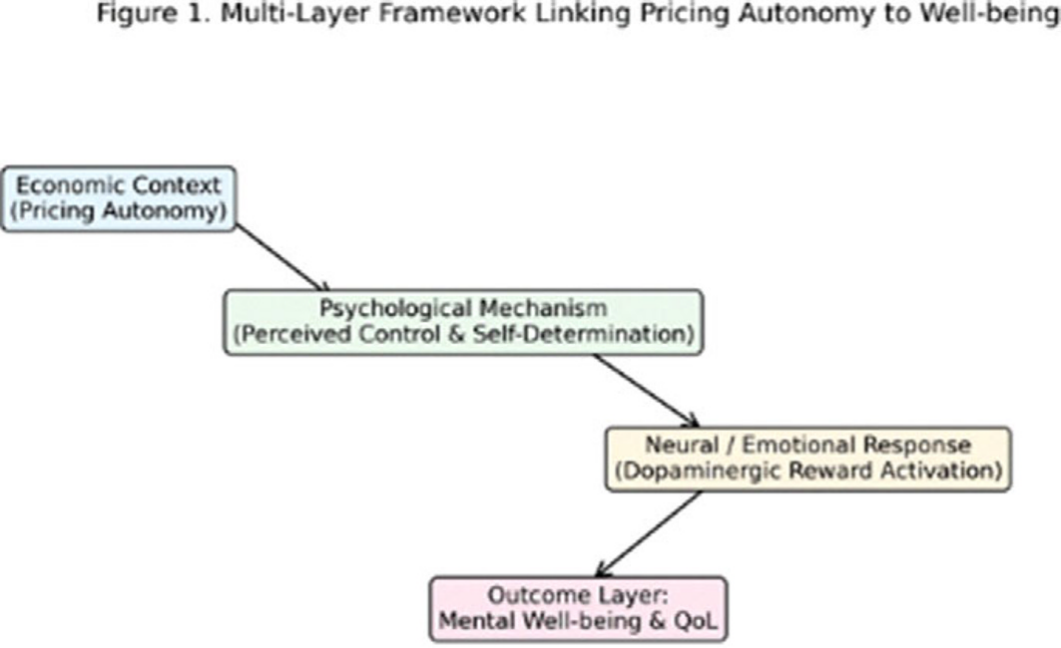

**Image 2:**
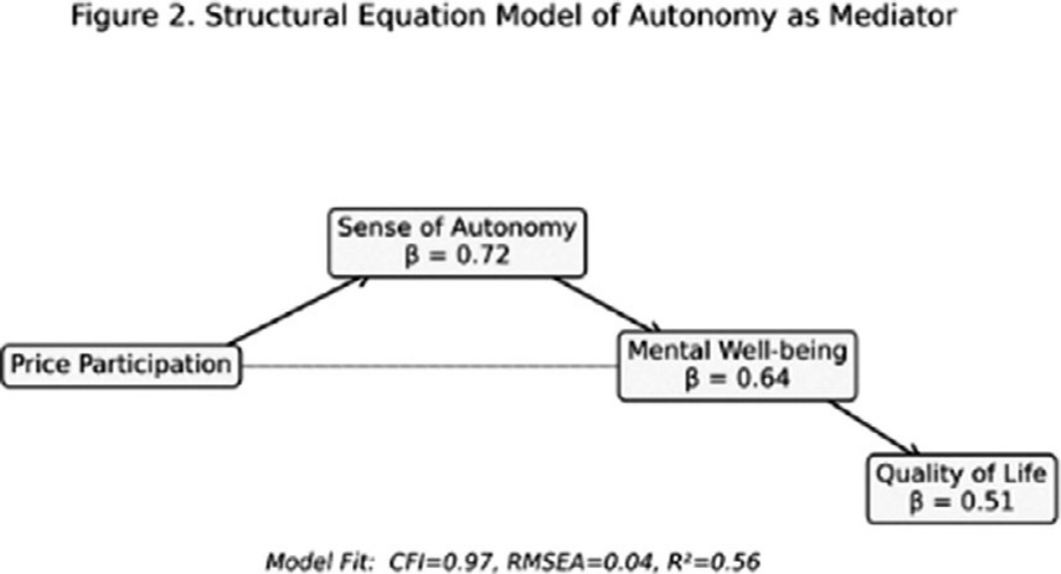

**Image 3:**
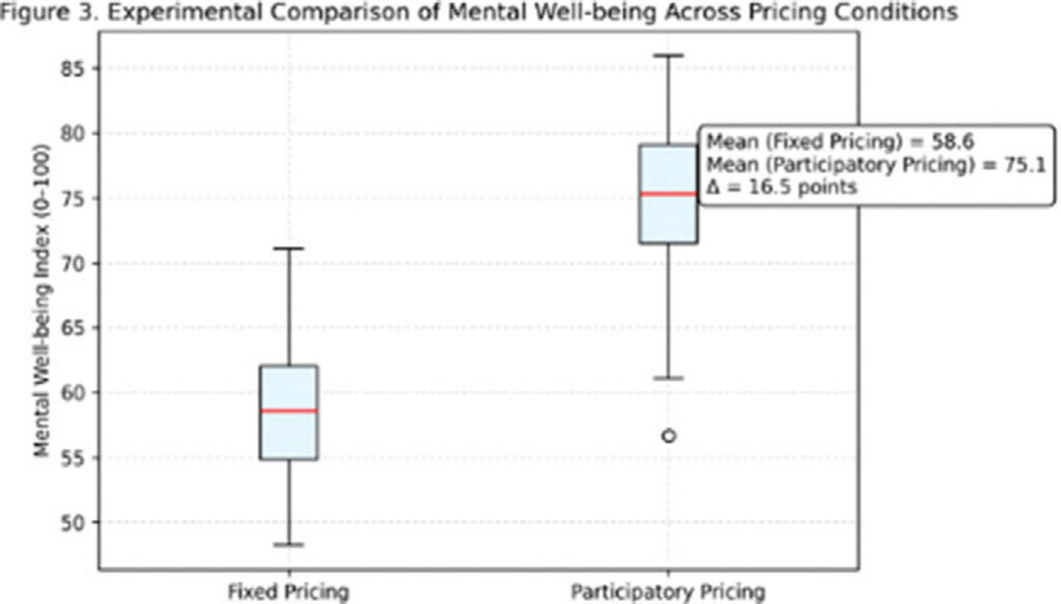
Image 3: Long description.

**Conclusions:**

The results indicate that even small opportunities for personal decision-making—such as self-determined pricing—can enhance the sense of autonomy and psychological well-being. From a psychiatric perspective, this behavioral moment reflects the therapeutic mechanisms of empowerment and agency, demonstrating that environments which strengthen decision-making freedom can subtly yet effectively promote mental health and quality of life. The present study bridges psychiatry and consumer behavior, suggesting that participatory models in markets and social institutions may function as subtle but effective psychosocial interventions for improving public mental health.

**Disclosure of Interest:**

None Declared

## Long descriptions

### Image 3: Long description

The y-axis is labeled Mental Well-being Index 0-100 with increments of 5. The x-axis has two categories.

1. Fixed Pricing on the left. The box plot shows a median red line at approximately 58.6. The interquartile range box extends from 55 to 62. Whiskers reach down to approximately 48 and up to 71.

2. Participatory Pricing on the right. The box plot shows a median red line at approximately 75.1. The interquartile range box extends from 71 to 79. Whiskers reach down to 61 and up to 86. There is a single outlier circle plotted at approximately 57.

A text callout box positioned to the right of the second plot provides the following data. Mean Fixed Pricing equals 58.6. Mean Participatory Pricing equals 75.1. Delta equals 16.5 points.

Navigate back to Image 3:.

